# A Qualitative Exploration of Quitline Perceptions among African American and Hispanic or Latino Adults Who Smoke

**DOI:** 10.1007/s40615-025-02600-9

**Published:** 2025-08-13

**Authors:** Sophia I. Allen, Candace R. Bordner, Gail D’Souza-Rushton, Nicolle M. Krebs, Andrea L. Hobkirk, William A. Calo, Kenneth R. Houser, Craig Livelsberger, Jonathan Foulds, Tilicea Henry, Caroline Eisele, Jessica Yingst

**Affiliations:** 1 Department of Public Health Sciences, Penn State Center for Research On Tobacco and Health, Penn State College of Medicine, 400 University Drive, Hershey, PA 17033 USA; 2 Penn State Cancer Institute, Hershey, PA 17033 USA; 3 Department of Medicine, Penn State College of Medicine, Hershey, PA 17033 USA; 4 Department of Nutritional Sciences, The Pennsylvania State University, University Park, PA 16802 USA; 5 Department of Psychiatry and Behavioral Health, Penn State College of Medicine, Hershey, PA 17033 USA; 6 Department of Public Health Sciences, Penn State College of Medicine, Hershey, PA 17033 USA

**Keywords:** African Americans, Disparities, Hispanic Americans, Quitlines, Smoking

## Abstract

**Introduction:**

Tobacco quitlines are efficacious, accessible, and cost-effective. While the prevalence of cigarette smoking remains higher among non-Hispanic White adults, the quit ratio has remained lower for African American (AA) and Hispanic or Latino (H/L) adults who smoke. The National Jewish Health Quitline (NJH QL) has sought to address whether current QL services provide effective and equitable tobacco cessation services. This study evaluated the experiences and perceptions of QL services among AA and H/L adults who smoke.

**Methods:**

Our team recruited adults who smoke for a qualitative study from July 2021 through October 2021 who had or had not engaged with QL services across 9 states. NJH QL clients shared their perceptions of QL services and experiences with quitting smoking; non-users of QL services shared in-depth responses on their knowledge, perceptions, and barriers to using a telephone QL service. Interviews were recorded, transcribed, and coded to identify key themes. Inter-coder reliability was calculated, and agreement (kappa > .80) was achieved.

**Results:**

Fifty-four eligible participants were recruited and thirty-five (64.8%) participants (21 AA, 14 H/L) across 5 states completed an interview. Themes included an awareness of media campaigns, importance of enthusiastic support from coaches, uncertainty about the effectiveness of QLs, appreciation of free nicotine replacement therapy and clear instructions for use, and an interest in tailored advertising and counseling by race or ethnicity matched coaches.

**Conclusion:**

The findings suggest that QL use among AA and H/L adults with historically low quit rates could be improved with tailored advertising and treatment conducted by enthusiastic and demographically matched coaches.

**Supplementary Information:**

The online version contains supplementary material available at https://doi.org/10.1007/s40615-025-02600-9.

## Introduction

Cigarette smoking remains a leading cause of morbidity and premature mortality in the United States [1]. Each year, most adults who smoke report wanting and attempting to quit [2, 3], with significant variations in quit interest between race and ethnicity. African American (AA) adults who smoke report greater desire to quit and more attempts to quit, compared to non-Hispanic White (NHW) smokers [4], while Hispanic or Latino (H/L) adults who smoke report less interest in quitting compared to AA and NHW adults who smoke [5, 6]. Despite interest, evidence suggests that AA and H/L smokers are less likely to successfully quit due, in part, to targeted marketing of tobacco products in neighborhoods with greater percentages of AA and H/L people [3]. These groups also have lower utilization of cessation treatments such as medication and/or counseling [7].

Telephone smoking cessation counseling services, generally known as “quitlines” (QLs) have become a key component of evidence-based smoking cessation treatment in the United States [8, 9]. Quitlines provide a variety of services including counseling by trained coaches, information on how to quit, referrals to health-related services and community group counseling, medications approved by the U.S. Food and Drug Administration (FDA), self-help materials, and technology-mediated services like web-based, texting and chat programs, smartphone applications, and social media [8, 9]. The services offered vary across states and U.S. territories.

The convenience and low cost of quitlines can provide benefits to those who are aware of the services, and awareness can vary in different regions of the U.S. and among minoritized populations. [10]. One study examining the California state QL found AA smokers were less aware of QL services than NHW smokers but once they were aware of such services, they were more likely to utilize them [11]. Under California’s Medicaid Incentives to Quit Smoking Program, another study found that African Americans and English-speaking Latinx who used the telephone-based counseling service through California’s Smokers’ Helpline had higher engagement with the financial incentive ($20 gift card) than White (non-Latinx) callers [12]. The Washington State QL found utilization rates and satisfaction ratings differed across AA and H/L or Latino smokers [13]. A study examining QL reach across 45 states by race/ethnicity found the overall annual average reach from 2011–2013 was 1.17% (Black), 0.87% (White), and 0.66% (Hispanic) [14]. QL services may be particularly appealing to racial or ethnic minorities due to convenience (i.e., flexibility, easy accessibility), free cost, and caller anonymity. However, barriers to QL utilization, such as mistrust in QL service providers among minorities [15] could counterbalance the advantages of state QL services and contribute to the overall low rates of use. Additionally, more research is needed to evaluate the differences in state-run QL utilization and effectiveness among racial and ethnic groups in the U.S. [16].

The purpose of this study was to evaluate perceptions of QL services among racial and ethnic minorities, particularly AA and H/L communities in select states. The National Jewish Health Quitline (NJH QL) is a multi-state QL operator that delivers services in 21 states [17]. NJH is contractually responsible for QL operational activity, including receiving and processing all intake calls, performing outgoing cessation counseling calls, the provision of nicotine replacement therapy (NRT) to qualified participants, and program evaluation and outcomes reporting. In this study, we aimed to assess the experiences of AA and H/L adults who smoke, or had recently quit, and evaluated their perceptions of smoking cessation QL services through qualitative interviews among select states who opted-in to provide quitline client data.

## Methods

### Design

In this qualitative interview study, participants were recruited over a 4-month period from July 2021 to October 2021 who had or had not engaged with QL services across 9 states. We interviewed individuals who engaged with the QL to explore barriers and facilitators of QL engagement and with individuals who had not engaged with the QL to explore factors related to perception and access. For users of the QL service, NJH staff sent a text message to users within their database who opted-in to being contacted about potential research opportunities. Participants met the following criteria: aged 21 +, identified as AA or H/L, and called the QL less than 6 months and more than 4 weeks prior to contact. The contact details of those who agreed to participate were passed on to Penn State researchers (regardless of the outcome of their quit attempt). To identify smokers who had not engaged with a QL (non-users), researchers utilized Amazon Mechanical Turk (Mturk). Mturk is a website run by Amazon that works as a readily available marketplace to match potential participants with surveys and other online jobs that they may be eligible to complete [18]. In addition, the participant database maintained by the Penn State Center for Research on Tobacco and Health (PS CRTH) was utilized for recruitment of smokers who had not engaged with a QL. Participants in this database who identified as AA or H/L adults were emailed a brief description of the study and invited to participate. This research was approved by the Penn State Institutional Review Board and informed consent was collected prior to participants starting the study.

Once potential participants were identified, they were sent a link to complete a short screening survey via REDCap (Research Electronic Data Capture). REDCap is a secure, web-based application designed to support data capture for research studies [19, 20]. Eligibility for the study was determined from the screening questions using the criteria listed in Table 1. Non-QL users were recruited from the same 9 states that the QL users were recruited from.

**Table 1:** Inclusion criteria to identify NJH Quitline users and non-users

NJH Quitline Users	NJH Quitline Non-users
21 years old or older	21 years old or older
African American or Hispanic/Latino	African American or Hispanic/Latino
Have called NJH Quitline for help to quit smoking in the past year^a^	Currently a daily cigarette smoker^b^
Completed at least 2 telephone calls with National Jewish Health^c^	Currently interested in quitting smoking in the next 12 months
Resided in CO, ID, MA, MI, NV, PA, UT, VT, WY	Resided in CO, ID, MA, MI, NV, PA, UT, VT, WY
Have tried to quit smoking for at least one day or longer in the past 12 months	Have tried to quit smoking for at least one day or longer in the past 12 months
First call was at least 4 weeks but less than 6 months ago	Have never called a telephone smoking cessation quitline for help to quit smoking
Access to a computer/smartphone and a reliable internet connection to complete Zoom meeting	Access to a computer/smartphone and a reliable internet connection to complete Zoom meeting
Able to speak, read, and write in English	Able to speak, read, and write in English

^a^Quitline users did not have to be successfully quit to participate in the study

^b^Currently a daily cigarette smoker = smoked at least 100 cigarettes in lifetime and who currently smokes cigarettes

^c^Completed assessment/intake call plus at least one coaching call

### Procedures

Potential participants (both NJH QL users and non-users) were screened for eligibility. During the screening survey, participants were asked to report demographic characteristics, including age, gender, race, and ethnicity. Participants were also asked questions [21] about their smoking history, including whether they used tobacco products in the past 30 days, the type of tobacco products they had used in the past 30 days, and the frequency of use. To measure participants quitting history and willingness to quit smoking, they were asked, “Have you tried to quit smoking for at least one day or longer within the past 12 months?” and “Would you like to quit smoking within the next 12 months?” Finally, to inquire if participants had ever used a QL service before, participants were asked, “Have you ever called a telephone “quitline” in order to get telephone help to quit smoking?” If they answered yes, they were asked how many times they contacted the quitline and how long ago they contacted the quitline. Participants were also asked in which state they resided and if they had a reliable phone and internet connection so they could participate in the Zoom (Version 5.7.0) interview.

Eligible participants identified from the screening survey were contacted via telephone to set up a day/time to complete informed consent and the qualitative interview on Zoom (if agreed to participate). During the screening and enrollment processes some participants could not be contacted or did not show for their interview and so those who completed interviews were less than expected, which reduced the number of participants from each of the states that opted-in to provide their data. The Zoom interview was a semi-structured interview with a series of probing questions for each group (Online Resource 1). The NJH QL participants were encouraged to share their smoking history, prior experiences with quit attempts, and experiences and perceptions of the NJH QL service. Non-users of a QL were asked to elaborate on their knowledge, perceptions, and barriers to using a telephone QL service. After the conclusion of the interview, the participants were compensated with a one-time payment of $100 via an electronic Amazon gift card. Each interview lasted approximately one hour and was audio and/or video recorded via Zoom. Once these interviews were completed, the primary endpoint of the study were the narratives of the participants. The recordings of the interviews were transcribed using Zoom and reviewed by researchers (SA, CB, GD, and JY).

### Data Analysis

Means and frequencies were used to quantitatively analyze the participants overall and by group (user or non-user). Sample sizes varied because of missing data for three participants who reported they had quit at the time of the interview and therefore some questions did not apply to these individuals. We used a thematic analysis approach to explore perceptions of using Quitline services among QL users and non-users. Qualitative data analysis followed standard methods as recommended by Patton [22]. Patton suggests inductive coding, which is the process of reading transcripts repeatedly to allow major themes to emerge. The study team utilized this method to generate two separate codebooks, one for QL users and one for non-users. Researchers (SA, CB, GD, NK, and JY) were involved during the data analysis process. First, researchers (SA, CB, GD, and JY) independently reviewed the interviews and generated preliminary codes based on the interview data, with JY assigned as the main codebook editor. Then, researchers met to discuss the independently developed codes and compile the final codebooks. All interviews were then imported to NVIVO 12 software (QSR International, Burlington, MA, USA) for coding. Researchers (SA, CB, and NK) first coded 25% of the interview data for each group. Inter-coder reliability was then calculated, and agreement (kappa > 0.80) was achieved for each codebook separately) [23, 24]. Finally, researchers (SA, CB, and NK) coded the remaining data.

## Results

Overall, 377 participants were screened for the study. Of those screened, 86 (22.8%) were eligible with 62 participants from NJH QLs and 24 non QL users from both Mturk and the PS CRTH. Among those eligible, 83 (96.5%) were able to be contacted, 54 (65.1%) scheduled an interview, and 35 (64.8%) completed an interview across 5 states (Table 2). Participants who completed the study (n = 35) had a mean age of 49.9 years (SD = 12.2, Range = 26–70), were 62.9% (n = 22) female, 65.7% (n = 23) were AA, and 28.6% (n = 10) were H/L. One individual identified as both AA and H/L (2.9%). Although some participants used the NJH QL for help with quitting smoking, at the time of their interview the majority (91.4%) of the participants reported current use of cigarettes, with 82.9% reporting daily use and an average of 14.8 (SD = 8.2, Range 4–40) cigarettes per day (CPD), and 3 had successfully quit smoking. Demographic data for NJH QL users and non-users are presented in Table 2.

**Table 2:** Characteristics of participants by Quitline Use Status

	NJH QL Users (n = 21)	Non-users^a^ (N = 14)
Mean age in years (SD, age range in years)	51.7 (12.0, 30–70)	47.2 (12.4, 26–66)
Female, % (n)	71.4 (15)	50.0 (7)
African American, % (n)	71.4 (15)	64.3 (9)
Hispanic/Latino, % (n)	28.6 (6)	35.7 (5)
Used tobacco in the past 30 days, % (n)		
Cigarettes	85.7 (18)^b^	100.0 (14)
Cigars	14.3 (3)	7.1 (1)
Pipes	0.0 (0)	7.1 (1)
Chew	4.8 (1)	0.0 (0)
Electronic cigarettes	11.1(2)	0.0 (0)
Mean number of cigarettes per day (SD, range)	12.8 (7.5, 4–35)	17.3 (8.6, 5–40)
Smokes Everyday, % (n)	83.3 (15)	100.0 (14)
Smokes Some days, % (n)	14.3 (3)	0.0 (0)
Have tried to quit in the past 12 months, % (n)	100.0 (18)^b^	100.0 (14)
Interested in quitting in the next 12 months, % (n)	100.0 (18)^b^	100.0 (14)
Ever used quitline service, % (n)	100.0 (21)	0.0 (0)
Used quitline service in the past 12 months, % (n)	100.0 (21)	0.0 (0)
Number of calls with the Quitline, % (n)		
2 or 3 times	47.6 (10)	—
4 or 5 times	52.4 (11)	—

— = not applicable; *SD* = standard deviation. “Current cigarette smokers” = smoked at least 100 cigarettes in lifetime and who currently smokes cigarettes

^a^Non-quitline users had to be current cigarette smokers

^b^Three participants were excluded from analysis because they had quit smoking at the time of the interview. Participants resided in the following states: Colorado (5 H/L), Massachusetts (1, AA), Michigan (8; 1 H/L, 7 AA), Nevada (1, AA), Pennsylvania (20; 14 AA, 6 H/L)

Key themes were identified from interviews with both the NJH QL users and non-users. Subthemes were identified and supported with quotes from participants. A list of all participant quotes categorized in subthemes is included in Online Resource 2.

## Quitline Engagement

### Sources of knowledge about the Quitline

Many participants who used the QL heard about it or saw advertisements (ads) from several sources including television and on social media (e.g., Instagram, Facebook). One participant stated, “Those commercials are excellent, every time they came on, I was saying that’s me, that’s what I need to do. I need this. Maybe three or four times of seeing the commercial and then I went ahead and called.” Participants also reported hearing about the QL from their healthcare provider and from their family and friends. One participant said, “I saw it on Instagram…and I was like, okay, that sounds like a good idea.” “My doctor actually had told me about the Quitline, and I decided to try it out.”

### Reasons for not engaging with the Quitline

Among non-users of the QL, some participants said they did not pay attention to the commercials they saw, fast-forwarded through commercials on streaming services, or were unsure that the counseling component would help them to quit. Some participants reported lack of trust as a barrier to calling the QL and suggested that callers should be given time to learn about the program before agreeing to participate. One participant discussed how the lack of trust of the government due to the historical harm of AAs in health research can be one reason for not engaging with the QL,

“…you need to find the way that you can say listen we’re not here to dupe you, we are not with the government, we’re not going to do an experiment. This is information we just want to share with you, and if you find this information useful, you take the time to give us a call back and then we’ll go ahead and continue.”

Another participant stated, “I’d rather call someone that I knew…if I was struggling…instead of calling a hotline.”

The content of the ads was another reason participants did not call the QL. They disliked the content of the ads and felt the content was trying to make them feel guilty about their smoking and inflict fear about conditions that may arise if they do not quit. One participant suggested to have less harsh messaging, to include information on eating healthy instead of smoking, and the use of NRT, like the gum, to help during a quit attempt. Other individuals suggested that they did not call the QL because they did not perceive that they needed help, or that they were not ready for help. One participant stated, “I always felt like I had it under control.” Another stated, “I wasn’t interested in quitting at that point.”

### Suggestions to improve advertising and reach

Participants said that while the QL currently does a lot of advertising, individuals who smoke still did not know it existed. Participants suggested the QL increase the number of ads and broaden the types of ads that are used to engage with individuals who need help to quit smoking. For example, ads on social media or billboards would let individuals know that there is help available on how to quit smoking. Other suggestions included putting signs in yards, making phone calls, sending text messages, promoting more on TV, and walking door-to-door and passing out flyers.

## Perceptions of coach support

### Positive perceptions of coaching

Participants reported positive aspects about the support and encouragement they received from the QL. One participant was appreciative of having a listening ear who understood the difficulty of quitting. Another participant stated, “…they were really reassuring, kept me on track, let me know I wasn’t in this alone, so I really appreciated that.” One participant liked how the coaches made them feel comfortable with no judgement, stating, “They make you feel really comfortable…there’s no judgement, but there is accountability, so it feels good.” Both QL and non-QL users said that they appreciated the actual and perceived benefit of having the QL hold them accountable.

### Negative perceptions of coaching

In addition to the concerns of some participants who never called the QL, those who did call also questioned whether the counseling they received was a helpful way to quit smoking. One participant thought that the coaches were not engaged enough and that they were not providing enough encouragement. Finally, some participants felt that based on their interactions, the coaches may need more training. One participant said, “It seemed like an obligation for both of us, for myself, and the quit coach. And we would both rather be doing other things.”

### Suggestions to improve coach support

Participants provided several recommendations to improve the level of support provided. Other participants suggested that matching coaches and callers by race and ethnicity would improve the level of support felt. One participant said, “I’d be more open to a conversation with Tyrone than I am to a conversation with Chad.”

Other individuals suggested that pairing the same coach and caller each time would be beneficial. A stricter calling schedule could ensure matching with the same coach. In scenarios where callers could not be matched with the same coach, the coaches could ask a couple more personable questions to demonstrate that they know a little more about the caller than just their smoking goals. Participants also suggested that avenues, such as through an app or chat, to connect with coaches other than via telephone would be beneficial. One participant suggested incorporating online resources to help people to stop smoking.

## Perceptions of Quitline benefits

### Education

Participants commented that the education provided by coaches was beneficial. Several participants commented that the coaches educated them and helped them to develop a plan to combat the difficulties associated with quitting. Participants liked the strategies provided by the coaches and the education about the harms of smoking. Participants were emailed self-help materials that could be printed out.

### Smoking Cessation Medication

Participants thought that providing medication was a great benefit of utilizing the QL, particularly for those with insurance that did not cover smoking cessation medications. One participant mentioned, “They sent me the patches and I appreciate it, because they were free, ‘cause for me with my insurance, I have to pay $50.” Another participant also commented how the upfront cost of nicotine replacement was a deterrent for use, so being provided the medication at no cost to callers was the reason for use,

“…like Nicorette is expensive, not that cigarettes are inexpensive, mind you, it’s the initial upfront cost… a pack of Nicorette, oh $40, well screw it I’ll just get a pack of cigarettes for $10 which is stupid, you know what I mean that we would think like that, but I mean that’s how people think it’s that upfront costs…”

Participants appreciated the option to select the nicotine replacement products that they wanted to use.” One participant stated, “I like how you can choose. They don’t say we get it, we’re going to give you patches…you have a choice what you would like.” They also appreciated the information their coach was able to provide about using the medication. One participant stated, “…we talked about like using half the patch…only peeling off part of it because I felt like the dose was too strong.”

## Perceptions of Quitline Resources

### Suggestions for improvement

Participants made several suggestions to improve the resources provided by the QL and the delivery of these resources. Some participants reported confusion and frustration about receiving the nicotine replacement medications while others did not receive the resources or medications at all or in the timeframe that they were expecting. Suggestions were made to ensure that the QL “…keep with their promises” and to offer an option to mail resources to users who may not have a printer. One participant stated,

“So I’m a pen and paper kind of person, like it’s real when I touch something and write something, but like some worksheets to print off if you have a printer just feels very, you know, inaccessible to a lot of folks, you know, if you want to think about the demographic this is supposed to be reaching like Black and Brown communities, etc. Like, not to say that Black and Brown people don’t have printers in their homes, but when you look at how the disproportionate amount of people that smoke that are also impoverished chances are [a] printer might not be at the top of their list, you know.”

Importantly, the lack of clarity about medication instructions and delivery delays resulted in one participant not using the medication at all. Finally, participants suggested providing additional resources for conditions related to smoking to include stress and mental health. One participant stated, “So if you are going to take the time to zero in on a community, take into account all the different health issues that a community might experience and combine that in your treatment of the person.” Another participant discussed stressors and mental health support by stating, “A lot of people associate smoking with stress…talk with the community about the stressors that are making them smoke, it’s going to open up another channel for people to feel more comfortable to talk about the things that are actually going on in their life…turn into more of mental health support then just quit smoking cigarettes.”

## Discussion

In this study, we assessed the experiences of AA and H/L individuals and evaluated their perceptions of QLs in general. Those who used the QL heard about it from several sources including TV, social media, their health care provider, and family and friends. We found that many participants who used the QL had positive feedback about the support and encouragement they received. The counseling kept them accountable, and they appreciated the benefits offered such as the education on the harms of smoking and the option to choose their preferred NRT. However, some participants who had used a QL experienced confusion and frustration in the timing of delivery of NRT and with the printing of self-help materials for those who may lack the resources like a home printer or broadband access.

### Quitline Reach

Participants made several comments and suggestions regarding ways to increase reach with the QL. First, participants suggested that marketing materials be inclusive and include representations of all ages. Participants reported that they wanted to see people like them in the ads. This finding is supported by previous research which shows that the marketing of smoking cessation campaigns to racial and ethnic minorities is more successful when materials are designed to be racially and ethnically diverse. Another study also mentioned that campaigns should be focused on media platforms (Facebook, Instagram, TV) that are designed to reach racial and ethnic minorities [25–27].

Participants also mentioned that people may not watch commercials now that they are able to fast forward or stream TV without commercials. Given this shift, it could be beneficial to start transitioning ads to other avenues like social media or online streaming services. There is evidence which suggests that AA and H/L populations are more likely to use streaming services than cable or satellite [26–28], showing the growing importance of focusing on avenues other than traditional cable television advertising. Many of the popular social media platforms (e.g., Facebook, Instagram), streaming services (e.g., Netflix, Hulu, Amazon, Prime Video), and ad-supported video-on-demand platforms (e.g., Tubi, PlutoTV, YouTube) include brief commercials that could be a potential location to place ads to reach the populations of interest.

Participants also thought that the current QL media campaigns overused scare tactics. A meta-analysis on fear appeals in public health campaigns suggest that fear-based health messaging only leads to health behavior change if the messaging is also effective. Ineffective fear-based messaging can cause viewers to become defensive and avoid change [29]. Effective messaging helps the viewer to believe that that they can take the suggested action (self-efficacy) and that the suggested action will help them to avoid the negative consequences. Thus, QL ads that provide information about smokers’ susceptibility to smoking-related harm and death should be supported by encouragement that the QL is effective in helping callers quit, clear steps on how to access the QL, and evidence-based information on how quitting can reduce the risk of illness and death for smokers.

While marketing of the QL through media campaigns is important, we also identified that many participants hear about the QL via recommendations from their providers. In addition to increasing media campaigns, efforts should be made to increase marketing of the QL to healthcare providers and to facilitate electronic referrals directly from the patient’s electronic medical record (with consent). Referrals or warm handoffs from physicians may also prove beneficial because it may increase the provider’s accountability for patients who express intentions to use the QL and will help facilitate troubleshooting of problems [30–32].

Doubt about the helpfulness was a barrier for those who have never called the QL, a finding that has been previously reported [33, 34]. To improve the perceived helpfulness and credibility of the QL, future marketing campaigns for both the public and healthcare providers should consider integrating real life success stories and highlight the training of the coaches as tobacco treatment specialists [35]. In addition, the ads should emphasize how the services include help with planning to quit, addressing barriers, and that callers could be eligible to receive free smoking cessation medications [35].

Some of the callers who used the QL mentioned promoting the QL services and benefits within their own community. These callers could partner with the QL to develop a community program using ambassadors or navigators who would be culturally matched to communities to promote QL services. Some AA and H/L smokers spoke of mistrust of institutions. A previous study found targeted outreach to AA and Latinx populations offering a financial incentive were successful in helping to eliminate barriers to counseling and cessation [12]. Establishing ambassadors or navigators among those who have used the QL in their journey to smoking cessation would overcome some of these cultural barriers [36].

### Quitline Engagement

Some participants mentioned that they felt the calls were impersonal and they wanted to feel more connected with their coach. Participants suggested that this could be achieved through asking some questions about the caller not related to smoking. This information could be added to the caller’s profile for other coaches to see and make a quick personal connection to the caller if it is the coach’s first time talking to the participant. Participants also suggested to use the same coach when possible because it would increase the connection with the caller and coach. Lastly, attempting to culturally match the caller to the coach would increase cultural sensitivity and the counseling experience. This could increase engagement and knowledge of the social contexts and stressors that callers from different racial and ethnic backgrounds experience.

Participants discussed the use of other technology platforms, such as text messaging or online chat, to connect with coaches. In the post-COVID era, telehealth through visual communications has expanded and may facilitate a better caller-coach relationship. In areas of behavioral health and substance use treatment, utilization of telehealth is even higher than in other areas of medicine [37, 38]. Previous barriers to telehealth, such as technology comfort level of users and regulations allowing use of virtual communications for healthcare, have diminished since the pandemic, providing an opportunity to incorporate this technology to QL services.

### Improve Resources

QLs could improve the usability of their resources by providing callers with the option to receive their resources in a printed form, versus online. A recent poll by Pew Research found that 96% of U.S. adults (N = 5,626) say they use the internet. However, 79% subscribe to home broadband internet service. Among these 73% of AA and 73% of Hispanic adults have access to home broadband internet service, compared with 83% of NHWs [39]. Additionally, 91% of U.S. adults say they own a smartphone, with 91% who are NHW, 87% who are AA, and 93% who are Hispanic. Among these, 15% of U.S. adults are smartphone dependent and do not subscribe to a home broadband internet service; 22% of Hispanic adults and 19% of AA are smartphone dependent compared to 12% of NHW [39]. Furthermore, variations were seen in health information technology (HIT) usage across race and Hispanic-origin groups in a sample of 14,020 adults ages 18 or older. In the National Health Interview Survey (NHIS) data collected from July to December 2022, White adults had higher rates of HIT usage in the past 12 months to look for health or medical information (63.4%) compared to AA (49.0%) and Hispanic (46.2%) adults [40]. Therefore, QLs should consider internet usage and access of enrollees to their services when providing support.

QLs should provide clearer communication about medication delivery. Since participants call the QL when they are highly motivated, it is more helpful if medication is received quickly while callers are still in the same mindset and the motivation is still high. Delays or confusion about medication use can result if the caller receives the medication late.

Finally, offering resources for other health concerns may be beneficial. Participants acknowledged that smoking is related to several other health conditions, particularly mental health. They suggested the QL could provide additional resources for related conditions and perhaps provide connections with other services.

### Limitations

The standard limitations of qualitative evaluations apply here. The sample of smokers interviewed was small and likely non-representative of all AA or H/L smokers or QL users, therefore, we could not make any claims of significant differences in findings between these groups. Additionally, we only recruited H/L smokers who spoke English. As observed in the quotes from the interviews, interviewee opinions were not uniform and included some contradictory suggestions. Suggestions made by some of the participants included ideas that may already be implemented within QL services (e.g., advertising and materials including AA and H/L smokers, provision of online and texting services). Nonetheless, this study can be used as a basis to expand upon for future studies.

## Conclusions

This study evaluated smoking cessation QL services from AA and H/L smokers’ point of view. This information may help improve QL services in minoritized communities and help public health professionals increase health equity through smoking cessation interventions.

## Supplementary Information

Below is the link to the electronic supplementary material.

ESM1 (DOCX 25.7 KB)

ESM2 (DOCX 36.2 KB)
