# Supplementary material for: A Qualitative Exploration of Quitline Perceptions among African American and Hispanic or Latino Adults Who Smoke
Source: J Racial Ethn Health Disparities. 2025 Aug 13;13(6):4540–9. doi: 10.1007/s40615-025-02600-9 (PMC12372992; doi:10.1007/s40615-025-02600-9)
Supplement: Supplementary file 1 [file 40615_2025_2600_MOESM1_ESM.docx]

**Article Title:** A Qualitative Exploration of Quitline Perceptions among African American and Hispanic or Latino Adults Who Smoke

**Journal Name:** Journal of Racial and Ethnic Health Disparities

**Authors:**

Sophia I. Allen, Ph.D.^1,2§^; Candace R. Bordner, M.S.^3^; Gail D’Souza-Rushton, Dr.PH.^4^; Nicolle M. Krebs, M.S.^1^; Andrea L. Hobkirk, Ph.D.^1,5^; William A. Calo, Ph.D.^2,6^; Kenneth R. Houser, M.S.^1^; Craig Livelsberger, B.S.^1^; Jonathan Foulds, Ph.D.^1,5^; Tilicea Henry, M.D.^5^; Caroline Eisele, M.D. ^5^; Jessica Yingst, Dr.PH.^1^

**Author Affiliations:**

^1^Penn State Center for Research on Tobacco and Health, Department of Public Health Sciences, Penn State College of Medicine, Hershey, PA, 17033, USA

^2^Penn State Cancer Institute, Hershey, PA, 17033, USA

^3^Department of Medicine, Penn State College of Medicine, Hershey, PA 17033, USA ^4^Department of Nutritional Sciences, The Pennsylvania State University, University Park, PA 16802, USA

^5^Department of Psychiatry and Behavioral Health, Penn State College of Medicine, Hershey, PA, 17033, USA

^6^Department of Public Health Sciences, Penn State College of Medicine, Hershey, PA, 17033, USA

^§^Corresponding author contact information: Penn State Center for Research on Tobacco and Health, Department of Public Health Sciences, Penn State College of Medicine, MC CH69, 400 University Drive, Hershey, PA, 17033, USA; (717) 531-8521; [sia3@psu.edu](mailto:sia3@psu.edu)

**ORC IDs:** SIA: 0000-0002-1809-3749; CRB: 0000-0002-1087-2916; GDR: 0000-0001-5307-7771; NMK: 0000-0002-5824-4572; ALH: 0000-0002-3020-3963; WAC: 0000-0001-8452-5063; KRH: 0000-0003-4457-5853; CL: 0009-0004-6444-5102; JF: 0000-0003-2296-0726; TH: 0000-0002-1953-7081; CE: 0000-0003-4753-4803; JY: 0000-0003-3028-668X

**Supplementary Material: Interview Guides**

| **Participants who report having used the NJH Quitline:** | **Participants who report not having used the NJH Quitline:** |
| --- | --- |
| Thinking back to BEFORE you had contact with the quitline, tell me about your smoking. How long had you been smoking? How much were you smoking per day (CPD)? | Tell me about your smoking. How long have you been smoking? How much are you smoking per day (CPD)? |
| Still thinking back to BEFORE you had contact with the quitline, tell me about any attempts you had previously made to quit smoking. What methods did you use to quit? How long did the quit attempt it last? | Tell me about any attempts you have previously made to quit smoking. What methods did you use to quit? How long did the quit attempt last? |
| Thinking back to the FIRST TIME you had contact with the quitline, tell me about how you heard about the quitline. What made you decide to try it as a way to quit smoking? Did you have any concerns or doubts about whether it would be helpful? | This study is evaluating the effectiveness and reach of telephone support programs for quitting smoking. Can you tell me about any telephone support programs for quitting smoking that you know about? Where did you hear of them? What do you think about using a telephone support line for help to quit smoking?   - Have you ever heard of a free quit smoking service on the telephone (sometimes called the quitline or 1-800-QUIT NOW)? What do you think about using a telephone quitline to help you to quit smoking? - Have you ever seen any advertisements on the TV or heard them on the radio, in which it tries to encourage you to think of quitting, and gives a number to call? What did you think about that? - Have you ever received a handout or leaflet when you were seeing a healthcare provider that tries to encourage you to think of quitting, and gives a number to call? What did you think about that? |
| Still thinking back to the FIRST TIME you had contact with the quitline, you probably had to answer some basic questions about yourself to get registered before actually talking to a quit coach (smoking cessation counselor). Could you describe the process of getting registered? What were you asked? How long did it take? Describe your experience with getting started with your reason for calling… to get help to quit smoking? | What if I were to tell you there is a free telephone counseling service, in which a trained quit coach will talk to you on the phone, help you organize a quit attempt, mail free nicotine gum or patches to you if you need them, and follow-up with you to see how you are doing, did you know that service exists? What do you think about that? |
| Once you were registered, tell me about your experience with the quit coach/counselor.   - Can you describe the calls that you had? What did you talk about? - How many times did you talk to a quitline coach/counselor on the phone? - At what point in that process did you actually try to quit smoking? - Did you decide to try to cut down gradually or quit all at once, and how did you come to that decision? - Did the quit coach talk to you about using medications like nicotine replacement therapy to help you quit smoking?   - If yes, tell me about your experience. What did the quit coach advise you to use? Did you use it?   - If no, what would you have liked to hear about the use of medications to quit smoking? - Describe whether you felt the Quitline services you received were helpful. Why or why not? | What concerns or hesitations would you have about calling a quitline for help to quit smoking? What might make you more likely to try a service like that? |
| Did you ever receive any information about quitting smoking in the mail or via e-mail from the quitline (after you made contact and were registered for counseling)? If yes, what did you receive, and was it helpful? | Do you have any concerns about whether a service like that would be helpful to an African American or Hispanic/Latino smoker? If yes, describe your concerns and the reasons for those concerns. |
| The quitline also has online internet components and can send text messages to help smokers to quit. What do you think about these methods to help smokers quit? Were you told about these options? Did you use them? What suggestions would you make to improve this service? | The quitline also has online internet components and can send text messages to help smokers to quit. What do you think about these methods to help smokers quit? What suggestions would you make to improve this service? |
| Would you recommend that your family, friends or colleagues use the quitline to get help to quit smoking? Why or why not? | Thinking of yourself and your own experience, and thinking about your friends and family who smoke who are interested in quitting someday, what do you think would help you or others to quit cigarettes? Can you tell me a bit more about that and how it compares with your faith and trust in the quitline services? |
| Can you tell me anything that you think the quitline might be able to do differently in order to be more helpful to people in your community (i.e., friends, family who smoke)? | Can you tell me anything that you think a telephone quitline might be able to do differently in order to be more helpful to people in your community (i.e., friends, family who smoke)? |
| Is there anything else you would like to tell me about quitting smoking or your use of the quitline service? Do you have any questions for me? | Is there anything else you would like to tell me about quitting smoking or your use of a telephone quitline service? Do you have any questions for me? |
