# Supplementary material for: A Qualitative Exploration of Quitline Perceptions among African American and Hispanic or Latino Adults Who Smoke
Source: J Racial Ethn Health Disparities. 2025 Aug 13;13(6):4540–9. doi: 10.1007/s40615-025-02600-9 (PMC12372992; doi:10.1007/s40615-025-02600-9)
Supplement: Supplementary file 2 [file 40615_2025_2600_MOESM2_ESM.docx]

**Article Title:** A Qualitative Exploration of Quitline Perceptions among African American and Hispanic or Latino Adults Who Smoke

**Journal Name:** Journal of Racial and Ethnic Health Disparities

**Authors:**

Sophia I. Allen, Ph.D.^1,2§^; Candace R. Bordner, M.S.^3^; Gail D’Souza-Rushton, Dr.PH.^4^; Nicolle M. Krebs, M.S.^1^; Andrea L. Hobkirk, Ph.D.^1,5^; William A. Calo, Ph.D.^2,6^; Kenneth R. Houser, M.S.^1^; Craig Livelsberger, B.S.^1^; Jonathan Foulds, Ph.D.^1,5^; Tilicea Henry, M.D.^5^; Caroline Eisele, M.D. ^5^; Jessica Yingst, Dr.PH.^1^

**Author Affiliations:**

^1^Penn State Center for Research on Tobacco and Health, Department of Public Health Sciences, Penn State College of Medicine, Hershey, PA, 17033, USA

^2^Penn State Cancer Institute, Hershey, PA, 17033, USA

^3^Department of Medicine, Penn State College of Medicine, Hershey, PA 17033, USA ^4^Department of Nutritional Sciences, The Pennsylvania State University, University Park, PA 16802, USA

^5^Department of Psychiatry and Behavioral Health, Penn State College of Medicine, Hershey, PA, 17033, USA

^6^Department of Public Health Sciences, Penn State College of Medicine, Hershey, PA, 17033, USA

^§^Corresponding author contact information: Penn State Center for Research on Tobacco and Health, Department of Public Health Sciences, Penn State College of Medicine, MC CH69, 400 University Drive, Hershey, PA, 17033, USA; (717) 531-8521; [sia3@psu.edu](mailto:sia3@psu.edu)

**ORC IDs:** SIA: 0000-0002-1809-3749; CRB: 0000-0002-1087-2916; GDR: 0000-0001-5307-7771; NMK: 0000-0002-5824-4572; ALH: 0000-0002-3020-3963; WAC: 0000-0001-8452-5063; KRH: 0000-0003-4457-5853; CL: 0009-0004-6444-5102; JF: 0000-0003-2296-0726; TH: 0000-0002-1953-7081; CE: 0000-0003-4753-4803; JY: 0000-0003-3028-668X

**Supplementary Material: Participant Demographics and Quotes by Themes/Subthemes**

| **Theme / Subtheme** | **Participant ID** | **Age** | **Sex** | **Race** | **Hispanic** | **Quitline User** | **Quotes** |
| --- | --- | --- | --- | --- | --- | --- | --- |
| Engagement with the Quitline | | | | | | | |
| Sources of knowledge about the Quitline | 99 | 52 | F | NA | Yes | Yes | “Those commercials are excellent, every time they came on, I was saying ‘that's me, that's what I need to do. I need this.’ Maybe three or four times of seeing the commercial and then I went ahead and called” |
|  | 44 | 43 | F | AA | No | Yes | “…I don’t know if it’s from live streaming service…but I can fast forward and I don't have to watch commercials anymore. So, it was like in your face all the time. I don't see that anymore, really” |
|  | 6 | 47 | M | AA  NA  W | Yes | Yes | “I saw it on Instagram…and I was like, okay, that sounds like a good idea.”  “My doctor actually had told me about the Quitline, and I decided to try it out.” |
|  | 191 | 59 | F | AA | No | Yes | “…they have a lot of quit smoking groups too and I had heard a couple people talking about the 1-800 Quitline so that's why I went there.” |
|  | 37 | 47 | F | O  W | Yes | Yes | “I heard about it through my doctor because I told my doctor I needed to quit.” |
|  | 223 | 42 | F | AA | No | Yes | “So my father, he had recommended me to it…that’s actually what helped him quit…” |
| Reasons for not engaging with the Quitline | 278 | 50 | F | AA | No | No | “I never really paid attention. I never really looked into that, you know?” |
|  | 298 | 37 | F | AA | No | No | “I have never used one [Quitline] in my time…I don't think I would use one in the future. I'm not a big talker and I don't think talking about why I need a cigarette…it’s not going to help me.” |
|  | 376 | 47 | F | O | Yes | No | “…what is talking going to do…what [about] my next situation…I don’t have a phone to call or I can’t call in the moment…I’m going to smoke.” |
|  | 347 | 63 | M | AA | No | No | “…I don’t really see how bad or good seeing someone on the phone would be. No real human interaction. I don’t see how that would help me, that’s probably why I haven’t tried it yet.” |
|  | 303 | 43 | F | AA | No | No | “I’d rather call someone that I knew…if I was struggling…instead of calling a hotline.” |
|  | 352 | 65 | F | AA | No | No | “…you need to find the way that you can say listen we’re not here to dupe you, we are not with the government, we’re not going to do an experiment. This is information we just want to share with you, and if you find this information useful, you take the time to give us a call back and then we’ll go ahead and continue.”  “The one with the voice box was a stop smoking commercial directed at stop smoking, but the harshness of it. The shock value of it really irritated me.” |
|  | 212 | 26 | F | AA | No | No | “…kind of traumatic…it’s the truth, but it’s hardcore.” |
|  | 278 | 50 | M | AA | No | No | “I always felt like I had it under control.” |
|  | 219 | 45 | F | AA | No | No | “I wasn’t interested in quitting at that point.” |
| Suggestions to improve advertising and reach | 44 | 43 | F | AA | No | Yes | “I would say that they need to do a lot more advertising, a lot of people don't know that Michigan has this. Wonderful quitline that this help is available like I’d like to see more advertisement maybe on social media or some billboards because A lot of people still small because they honestly don't know how to quit or that there's like some kind of help you know, most of the help that's out here for people who are you know alcoholics or on drugs but nicotine is a lot, you know one in the same because it's an addiction.” |
|  | 347 | 63 | M | AA | No | No | “…promote themselves, advertise more, get out into the community. Make it known that they are available and the help that they could provide. Put some posters up,” |
|  | 219 | 45 | F | AA | No | No | “Maybe put signs in the yard or call your phone or send you a text message or promoting more on the TV. Or do some handing out walking door to door to pass out flyers, maybe. Or some things like that.” |
| Perceptions of Coach Support | | | | | | | |
| Positive perceptions of coaching | 6 | 47 | M | AA  NA  W | Yes | Yes | “I believe the line is helpful because it’s nice to be able to talk to somebody.” |
|  | 29 | 62 | F | AA | No | Yes | “They really try to encourage you…they were really helpful…they continue to check on me.” |
|  | 83 | 58 | F | AA | No | Yes | “…they were really reassuring, kept me on track, let me know I wasn’t in this alone, so I really appreciated that.” |
|  | 44 | 43 | F | AA | No | Yes | “They make you feel really comfortable…there’s no judgement, but there is accountability, so it feels good.” |
| Negative perceptions of coaching | 141 | 31 | M | O  W | Yes | Yes | “Talking my way out of a nicotine addiction, I don’t know, it just doesn’t seem to really help.” |
|  | 18 | 47 | F | W | Yes | Yes | “I just don’t see the coaching as really helpful. I’ve done like a more in-person type of coaching and that was really helpful. That’s not the person I’m going to call when I having a hard time. I have friends and family to talk to in those times…it’s just hard to get like the connection.” |
|  | 120 | 38 | F | AA | No | Yes | “I called and I spoke to someone… I said, yeah, you know I quit, I quit smoking I haven't smoked since XY and Z. And there was this long pause and it just broke my whole spirit, because it’s like are you still typing the notes from the last thing I said…like what’s the point?” |
|  | 6 | 47 | M | AA  NA  W | Yes | Yes | “I felt like I was a nuisance to them like they were just, I don't know. They weren't supportive.” |
|  | 37 | 47 | F | O  W | Yes | Yes | “It seemed like an obligation for both of us, for myself, and the quit coach. And we would both rather be doing other things” |
| Suggestions to improve coach support | 6 | 47 | M | AA  NA  W | Yes | Yes | “It's always been my thought you never fully understand what a person is going through until you've walked a mile or two in their shoes” |
|  | 212 | 26 | F | AA | No | No | “I’d be more open to a conversation with Tyrone than I am to a conversation with Chad.” |
|  | 37 | 47 | F | O  W | Yes | Yes | It’s strange to have a different person call me.” |
|  | 120 | 38 | F | AA | No | Yes | “I think that they want to be so accessible to be like whenever you call we will have someone for you, but maybe there’s a little pushback where it’s like well look I’m next available on Thursday.” |
|  | 18 | 47 | F | W | Yes | Yes | “Either through an app or a chat or something like that…for when people do need someone to talk to. I think that would be more beneficial than, you know, taking a phone call” |
|  | 278 | 50 | M | AA | No | No | “Some online methods to help stop smoking. I think that's good, because people be online all the time, so I think that [would] help real good” |
| Perceptions of Quitline Benefits | | | | | | | |
| Education | 191 | 59 | F | AA | No | Yes | “They basically gave you the tools, that's what I liked about them, they gave you a lot of tools.” |
|  | 77 | 59 | F | AA | No | Yes | “I liked when the Quitline was telling me about different diseases and the chemicals in cigarettes and stuff like that.” |
| Smoking Cessation Medication | 29 | 62 | F | AA | No | Yes | “They sent me the patches and I appreciate it, because they were free, cause for me with my insurance, I have to pay $50.” |
|  | 169 | 45 | F | W | Yes | Yes | “Prior to this last time, I knew they had the tools available to me they were able to send me gum or patches or lozenges or whatever those are. So that was helpful because it was an added cost just because you know, like Nicorette is expensive, not that cigarettes are inexpensive, mind you, it’s the initial upfront cost… a pack of Nicorette, oh $40, well screw it I’ll just get a pack of cigarettes for $10 which is stupid, you know what I mean that we would think like that, but I mean that’s how people think it’s that upfront costs…” |
|  | 191 | 59 | F | AA | No | Yes | “I like how you can choose. They don't say we get it, we're going to give you patches…you have a choice what you would like.” |
|  | 120 | 38 | F | AA | No | Yes | “…we talked about like using half the patch…only peeling off part of it because I felt like the dose was too strong.” |
| Perceptions of Quitline Resources | | | | | | | |
| Suggestions for improvement | 190 | 69 | F | AA | No | Yes | “I think they did a good job, but yeah, they need to make sure that they keep…with their promises. And so that would be my suggestion, like the promise that okay, well we'll send out the medication this week and, no, it didn't come that week. It came about two weeks later, oh yeah, and so that was not good.” |
|  | 120 | 38 | F | AA | No | Yes | “I think they should have an op􏰀on, where you they can just send you a packet as well, because I know we're like saving the earth so maybe at request you know because I’m, I’m a writer. So I’m a pen and paper kind of person like it's real when I touch something and write something, but like some worksheets to print off if you have a printer just feels very you know inaccessible to a lot of folks you know if you want to think about the demographic this is supposed to be reaching like Black and Brown communities, etc. like, not to say that Black and Brown people don't have printers in their homes, but when you look at how the disproportionate amount of people that smoke that are also impoverished chances are printer might not be at the top of their list, you know. And if it's not a right on PDF if you're asking me to engage with the content. How am I supposed to engage with it? it can be also very discouraging if you can't access the resources, you know?”  “So if you are going to take the time to zero in on a community, take into account all the different health issues that a community might experience and combine that in your treatment of the person.” |
|  | 298 | 37 | F | AA | No | No | “A lot of people associate smoking with stress…talk with the community about the stressors that are making them smoke, it's going to open up another channel for people to feel more comfortable to talk about the things that are actually going on in their life…turn into more of mental health support then just quit smoking cigarettes.” |
